# Forced Vasohibin‐1 Expression Increases Paclitaxel Sensitivity of Ovarian Cancer by Inhibiting Microtubule Activity

**DOI:** 10.1002/cnr2.70100

**Published:** 2024-12-22

**Authors:** Takahiro Koyanagi, Yasushi Saga, Yoshifumi Takahashi, Kohei Tamura, Suzuyo Takahashi, Akiyo Taneichi, Yuji Takei, Hiroaki Mizukami, Hiroyuki Fujiwara

**Affiliations:** ^1^ Department of Obstetrics and Gynecology, School of Medicine Jichi Medical University Shimotsuke Tochigi Japan; ^2^ Division of Genetic Therapeutics, Center for Molecular Medicine Jichi Medical University Shimotsuke Tochigi Japan

**Keywords:** cell cycle, detyrosinated tubulin, ovarian cancer, paclitaxel, vasohibin‐1

## Abstract

**Background:**

Vasohibin‐1 (VASH1), an angiogenic inhibitor, exhibits tubulin carboxypeptidase activity, which is involved in microtubule functions. Paclitaxel, the core chemotherapeutic agent for ovarian cancer chemotherapy, has a point of action on microtubules and may interact with VASH1.

**Aims:**

To examine the influence of VASH1 on intracellular tubulin detyrosination status, cyclin B1 expression, and paclitaxel chemosensitivity using VASH1‐overexpressing ovarian cancer cell lines.

**Methods and Results:**

Gene‐transfected human ovarian cancer cell lines were subjected to western blot analysis. Western blot analysis of VASH1‐overexpressing ovarian cancer cells revealed upregulated expression of detyrosinated tubulin and cyclin B1 compared with control cells. By WST‐1 assay, paclitaxel chemosensitivity of VASH1‐overexpressing ovarian cancer cells was markedly enhanced compared with that of control cells, whereas there was no significant difference in chemosensitivity to cisplatin. The forced expression of VASH1 enhanced tubulin carboxypeptidase activity and increased cyclin B1 expression, resulting in augmented paclitaxel chemosensitivity in ovarian cancer cells.

**Conclusion:**

Ovarian cancer treatment strategies targeting VASH1 can potentiate the effects of conventional chemotherapy by inhibiting angiogenesis and regulating microtubule activity.

## 1 Introduction

Ovarian cancer remains the most lethal gynecological malignancy, with 314 000 cases and 207 000 deaths annually worldwide [1]. Most patients with ovarian cancer are diagnosed at an advanced stage because of the lack of symptoms at an early stage. The standard treatment strategies for advanced ovarian cancer are primary debulking surgery and adjuvant chemotherapy with a combination of platinum and taxane preparations, with initial complete response achieved in approximately 80% of patients. However, the antitumor effects are usually transient, and more than half of patients experience abdominal recurrence with low chemosensitivity, ultimately leading to cancer‐related death [2, 3]. Moreover, factors such as NADPH: quinone oxidoreductase 1 (NQO1) and Solute Carrier Family 7 Member 11 (SLC7A11, also known as xCT) increase the antioxidant defense of ovarian cancer cells, which is the primary reason for increased chemoresistance [4, 5]. Recently, novel molecular‐targeted agents including bevacizumab and polyadenosine diphosphate‐ribose polymerase (PARP) inhibitors, have been used clinically [6]. Although these drugs show some antitumor effects, some of their side effects and the associated economic burden represent emerging problems that are yet to be overcome. Therefore, novel therapeutic strategies are urgently required.

Sustained tumor angiogenesis stands as a hallmark of cancer. Angiogenesis is crucial for the transport of oxygen and nutrients to the tumor during tumor development. Therefore, angiogenesis inhibition has become an important strategy in the clinical management of many solid tumors [7]. The vasohibin family includes vasohibin‐1 (VASH1) and vasohibin‐2 (VASH2). VASH1 is an angiogenesis inhibitor derived from endothelial cells (ECs). We previously reported that VASH1 overexpression inhibited tumor growth and peritoneal dissemination by inhibiting tumor angiogenesis, thereby prolonging host survival in murine xenograft models of ovarian cancer [8, 9]. Some researchers have reported that VASH1 represents a useful clinical biomarker for metastasis and poor prognosis, and may be a potential therapeutic target in epithelial ovarian cancer [10, 11, 12].

Recent reports indicate that the vasohibin family exhibits tubulin carboxypeptidase (TCP) activity related to microtubule polymerization [13, 14]. Paclitaxel (PTX), a key chemotherapeutic agent for ovarian cancer, acts as a microtubule depolymerization inhibitor [2, 3] and may interact with the vasohibin family. However, to date, there has been no report on the relationship between the TCP activity of VASH1 and cancer progression or chemosensitivity.

Therefore, in the current study, we examined the influence of VASH1 on intracellular tubulin detyrosination status and PTX chemosensitivity using VASH1‐overexpressing ovarian cancer cell lines.

## 2 Methods

### 2.1 Cell Lines and Their Transfectants

The human ovarian carcinoma cell line SKOV‐3 was purchased from the American Type Culture Collection (Manassas, VA, USA). The human ovarian serous adenocarcinoma cell line SHIN‐3 was provided by its establisher [15]. The cells were cultured in Dulbecco's modified Eagle medium/F12 (DMEM/F12; Thermo Fisher Scientific Inc. Waltham, MA, USA) supplemented with 10% fetal bovine serum (FBS; Sigma‐Aldrich; Merck KGaA, Darmstadt, Germany), and 1% penicillin/streptomycin (Thermo Fisher Scientific Inc.) at 37°C in a humidified atmosphere under 5% CO_2_. Human VASH1‐expressing or luciferase (LUC)‐expressing plasmids were constructed as described previously [8, 9]. We established human VASH1 gene‐transfected SKOV‐3 cells (SKOV‐3/VASH1) and LUC gene‐transfected SKOV‐3 cells (SKOV‐3/LUC) in 2015 [8], and human VASH1 gene‐transfected SHIN‐3 cells (SHIN‐3/VASH1) and LUC gene‐transfected SHIN‐3 cells (SHIN‐3/LUC) in 2016 [9]. Gene transfection of each clone was confirmed by western blot analysis, as described in our previous reports [8, 9].

### 2.2 Western Blot Analysis

Tumor cells were seeded onto 6‐well plates at 5 × 10^5^ cells/well. After incubation for 24 h, the tumor cells were lysed using lysis buffer (1% NP‐40, 150 mM NaCl, 50 mM Tris–HCl, pH 8.0). The extracted proteins were mixed with 1% sodium dodecyl sulfate (SDS) sample buffer (10 mM Tris–HCl, pH 7.5, 150 mM NaCl, 1% SDS, and ethylenediamine tetraacetic acid‐free protease inhibitor cocktail) (Roche, Basel, Switzerland), separated by electrophoresis using 10% polyacrylamide gels, and transferred onto polyvinylidene fluoride (PVDF) membranes (Merck KGaA). The membranes were incubated at room temperature for 1 h in PVDF Blocking Reagent for Can Get Signal (Toyobo Life Science, Osaka, Japan), washed three times using Tris‐buffered saline‐Tween‐20 (TBS‐T), and then incubated at room temperature overnight with the antibodies shown in Table 1 diluted in Can Get Signal Immunoreaction Enhance Solution 1 (Toyobo Life Science). After the reaction, the membranes were washed three times with TBS‐T and then incubated with peroxidase‐labeled anti‐mouse or anti‐rabbit antibody (GE Healthcare Japan, Tokyo, Japan) in Can Get Signal Immunoreaction Enhance Solution 2 (Toyobo Life Science) at room temperature for 1 h. The membranes were washed three times with TBS‐T, incubated with ECL Prime Western Blotting Detection Reagent (GE Healthcare Japan), and imaged using a cooled charge‐coupled device system (LAS‐4000mini: GE Healthcare Japan). The relative protein expression level was compared to that of alpha tubulin or β‐actin as 1.0 using ImageJ software (NIH, Bethesda, MD, USA).

**TABLE 1:** Primary antibodies used in this study.

Antibody	Catalog no.	Company
Anti‐detyrosinated alpha tubulin rabbit polyclonal antibody	ab48389	Abcam, Cambridge, UK
Anti‐alpha tubulin mouse monoclonal antibody	sc‐32 293	Santa Cruz Biotechnology Inc., Dallas, TX, USA
Anti‐cyclin B1 rabbit monoclonal antibody	12 231	Cell Signaling Technology Inc., Danvers, MA, USA
Anti‐β‐actin rabbit polyclonal antibody	A2066	Sigma‐Aldrich; Merck KGaA

### 2.3 Colorimetric Assay

The sensitivity of cancer cells to PTX (Sawai Pharmaceutical Co. Ltd., Osaka, Japan) and cisplatin (CDDP) (Nichi‐Iko Pharmaceutical Co. Ltd., Tokyo, Japan) was examined using a colorimetric assay using the Premix WST‐1 Cell Proliferation Assay System (Takara Bio Inc. Tokyo, Japan). Tumor cells were exposed to each chemotherapeutic agent at concentrations of 1–128 nM (for PTX) or 1–32 μM (for CDDP) for 48 h. The viable cell count is presented as a percentage of the count of the untreated control. A dose–response curve was constructed, and the 50% growth inhibitory concentration (IC_50_) was obtained for each chemotherapeutic agent.

### 2.4 Statistical Analysis

Statistical analysis was performed using EZR software (Saitama Medical Center, Jichi Medical University, Saitama, Japan). Student's *t*‐test was used to compare the two groups. *p* values < 0.05 were considered to indicate a statistically significant difference.

## 3 Results

### 3.1 Tubulin Detyrosination Status

**FIGURE 1:**
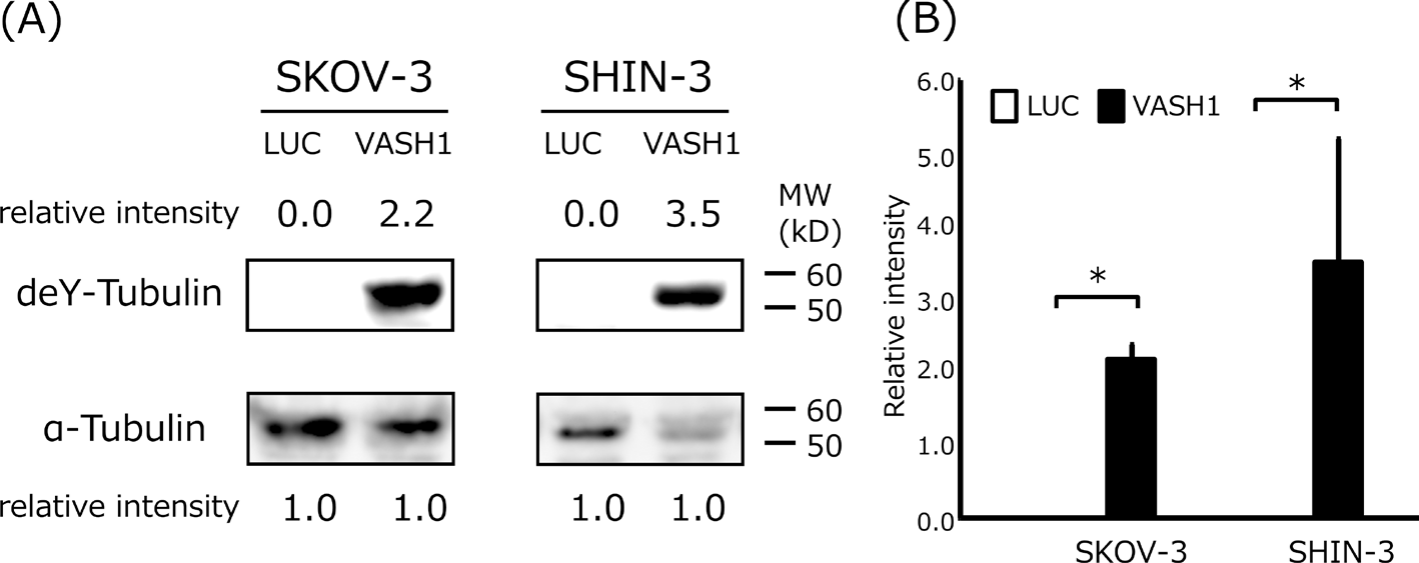
Western blotting of detyrosinated tubulin (deY‐tubulin) in VASH1‐overexpressing or control luciferase gene‐transfected ovarian cancer cell lines. (A) Control cells (SKOV‐3/LUC and SHIN‐3/LUC) very weakly expressed detyrosinated tubulin, while VASH1 gene‐transfected cells (SKOV‐3/VASH1 and SHIN‐3/VASH1) strongly expressed detyrosinated tubulin. α‐tubulin in the cell lysate was used as a loading control. Each number indicates the relative protein expression level compared with α‐tubulin as 1.0. (B) Densitometric analysis is shown in column graph. Data are shown as the mean and SD. *p < 0.05 (vs. LUC gene transfected cells). LUC: luciferase, VASH: vasohibin.

The results of the western blot analysis of VASH1 or LUC gene‐transfected control ovarian cancer cell lines are shown in Figure 1. Control cells (SKOV‐3/LUC and SHIN‐3/LUC) very weakly expressed detyrosinated tubulin, whereas VASH1 gene‐transfected cells (SKOV‐3/VASH1 and SHIN‐3/VASH1) strongly expressed detyrosinated tubulin. These results suggest that VASH1 possesses TCP activity and that its overexpression in ovarian cancer cells could induce strong tubulin detyrosination.

### 3.2 Cyclin B1 Expression

**FIGURE 2:**
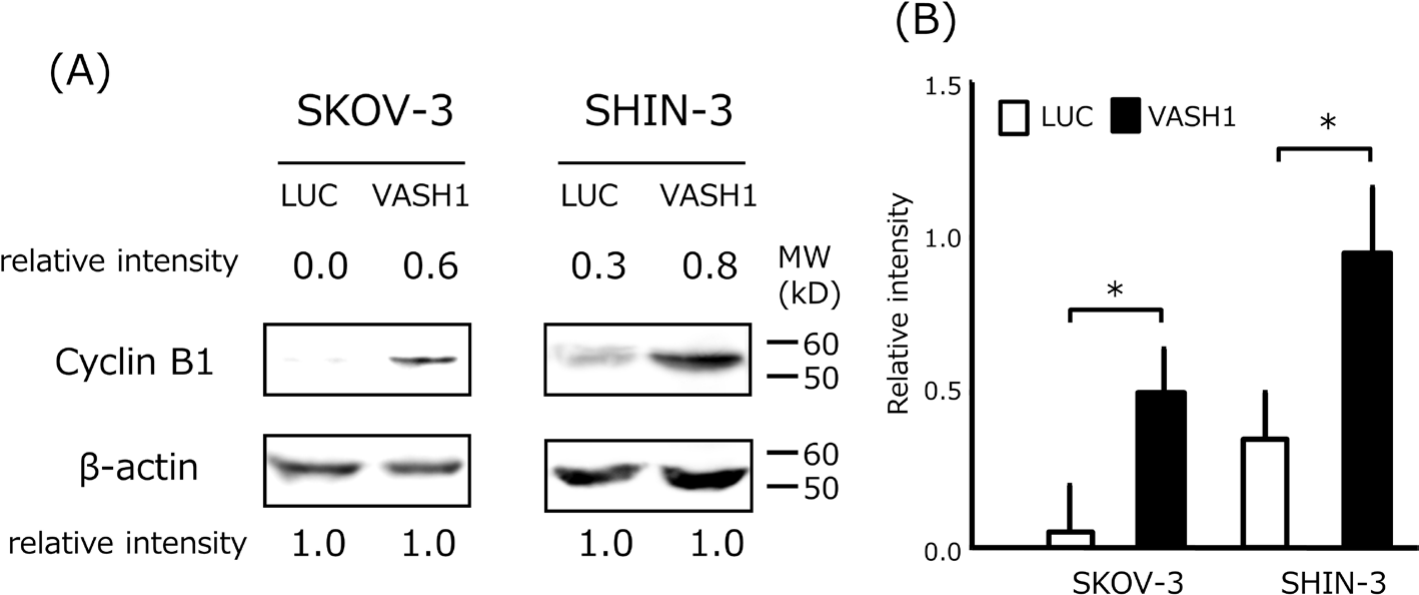
Western blotting of cyclin B1 in VASH1‐overexpressing or control luciferase gene‐transfected ovarian cancer cell lines. (A) Cyclin B1 expression was weakly detected in control cells (SKOV‐3/LUC and SHIN‐3/LUC), whereas SKOV‐3/VASH1 and SHIN‐3/VASH1 cells clearly expressed cyclin B1 at the position corresponding to a molecular weight of 55 kDa. β‐actin in the cell lysate was used as a loading control. Each number indicates the relative protein expression level compared with β‐actin as 1.0. (B) Densitometric analysis is shown in the column graph. Data are shown as the mean and SD. *p < 0.05 (vs. LUC gene transfected cells). LUC: luciferase, VASH: vasohibin.

Cyclin B1 expression was evaluated via western blot analysis. The control cells (SKOV‐3/LUC and SHIN‐3/LUC) showed weak cyclin B1 expression, whereas SKOV‐3/VASH1 and SHIN‐3/VASH1 cells clearly expressed cyclin B1 (Figure 2). These results suggest that VASH1 overexpression increased cyclin B1 expression in ovarian cancer cells.

### 3.3 Chemosensitivity

**FIGURE 3:**
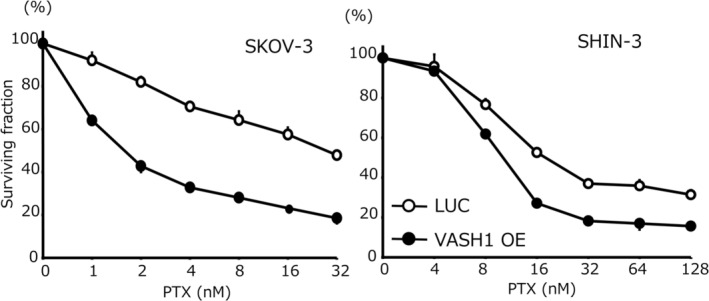
Chemosensitivity to PTX. The IC_50_ for PTX in SKOV‐3 was as follows: SKOV‐3/LUC, 28.4 ± 2.0 nM versus SKOV‐3/VASH1, 1.7 ± 0.1 nM (16.7‐fold higher sensitivity, *p* < 0.05). The IC_50_ for PTX in SHIN‐3 was as follows: SHIN‐3/LUC, 21.0 ± 0.1 nM versus SHIN‐3/VASH1, 10.7 ± 0.1 nM (2.0‐fold higher sensitivity, *p* < 0.05). Data are shown as the mean and SD (*n* = 3). LUC: luciferase, VASH: vasohibin, PTX: paclitaxel.

**FIGURE 4:**
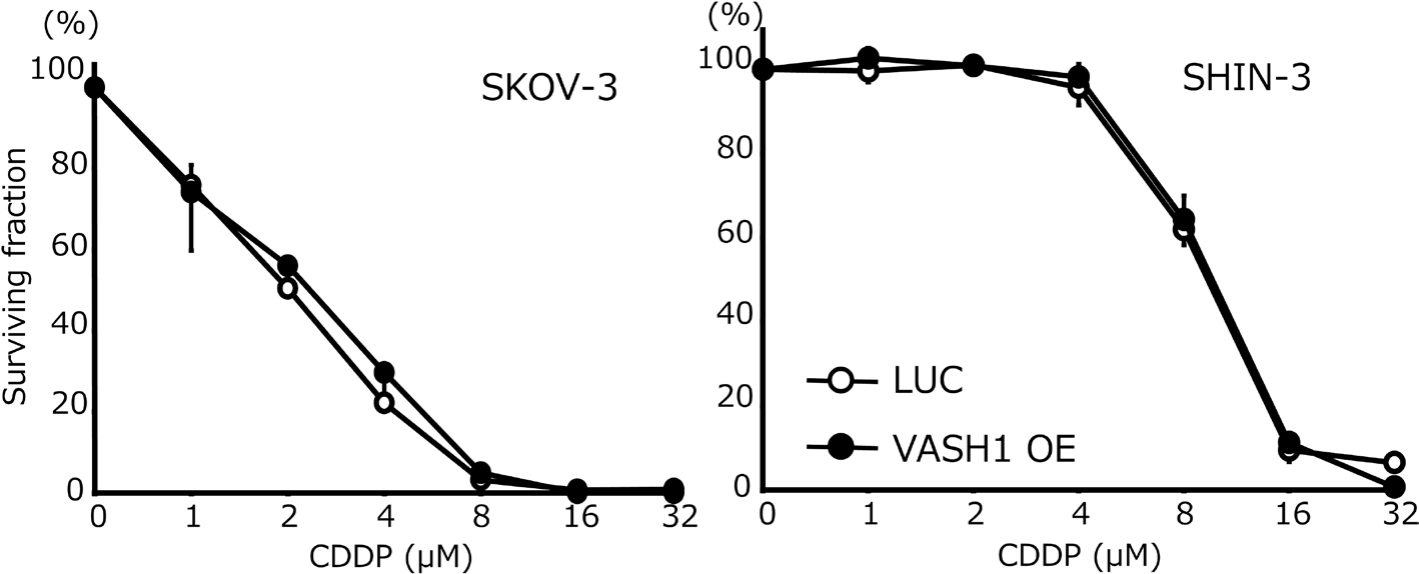
Chemosensitivity to CDDP. The IC_50_ for CDDP in SKOV‐3 was as follows: SKOV‐3/LUC, 2.0 ± 0.4 μM versus SKOV‐3/VASH1, 2.5 ± 0.4 μM (not significant). The IC_50_ for CDDP in SHIN‐3 was as follows: SHIN‐3/LUC, 9.8 ± 0.5 μM versus SHIN‐3/VASH1, 10.1 ± 0.7 μM(not significant). Data are shown as the mean and SD (*n* = 3). LUC: luciferase, VASH: vasohibin, CDDP: cisplatin.

The chemosensitivity of the examined cell lines to PTX is shown in Figure 3. The IC_50_ for PTX in SKOV‐3/VASH1 cells was 1.7 ± 0.1 nM, which was 16.7‐fold higher than that of control SKOV‐3/LUC cells (28.4 ± 2.0 nM) (*p* < 0.05). Similarly, the IC_50_ for PTX in SHIN‐3/VASH1 cells was 10.7 ± 0.1 nM, which was 2.0‐fold higher than that of control SHIN‐3/LUC cells (21.0 ± 0.1 nM) (*p* < 0.05). In contrast, as shown in Figure 4, no significant difference was noted in the IC_50_ for CDDP in the SKOV‐3 clones (2.5 ± 0.4 μM for SKOV‐3/VASH1 cells vs. 2.0 ± 0.4 μM for control SKOV‐3/LUC cells). Similarly, no significant difference was noted in the IC_50_ for CDDP in the SHIN‐3 clones (9.8 ± 0.5 μM for SHIN‐3/VASH1 cells vs. 10.1 ± 0.7 μM for control SHIN‐3/LUC cells). Collectively, these results demonstrated that the overexpression of VASH1 increased chemosensitivity to PTX, but not to CDDP, in ovarian cancer cells.

## 4 Discussion

In this study, forced expression of VASH1 was found to increase intracellular tubulin detyrosination and cyclin B1 expression. Furthermore, VASH1 overexpression significantly augmented sensitivity to PTX, but not to CDDP, in ovarian cancer cell lines. These results suggest that VASH1 affects microtubule activity and may represent a promising target for augmenting the chemosensitivity of ovarian cancer cells to conventional chemotherapy.

In the regulation of angiogenesis, VASH1 is mainly introduced in vascular ECs at the termination zone by angiogenesis simulators such as vascular endothelial growth factor (VEGF) and basic fibroblast growth factor (FGF), and inhibits angiogenesis by acting as a negative feedback regulator [16]. We previously reported that subcutaneous or intraperitoneal transplantation of ovarian cancer cells overexpressing VASH1 retarded tumor growth and peritoneal dissemination by inhibiting tumor angiogenesis, thereby prolonging host survival in murine xenograft models [8]. VASH1 has also been shown to inhibit the proangiogenic actions of FGF and platelet‐derived growth factor (PDGF) families [16]. Angiogenic factors other than VEGF are associated with resistance and tolerance to anti‐VEGF therapy [17]. A previous study demonstrated that VASH1 overexpression inhibited tumor growth and angiogenesis in an anti‐VEGF therapy‐resistant, PDGF‐producing ovarian cancer animal model [9]. Treatment with VASH1 may be effective for chemotherapy‐resistant ovarian cancer and may prevent adverse events of anti‐VEGF antibody therapy, such as hypertension and proteinuria, because VASH1 is usually induced as negative feedback regulator under conditions of abnormal angiogenesis, such as that within the context of tumorigenesis. Moreover, no obvious abnormality was observed in mice administered high‐dose VASH1 via an adenovirus vector [18].

VASH2, detected and isolated as a homolog of VASH1, inversely acts as an angiogenesis stimulator [19, 20]. VASH2 expression has been reported in various cancer types, where it functions to stimulate intratumoral angiogenesis in a paracrine manner [21, 22]. The administration of siRNA targeting VASH2 or neutralizing anti‐VASH2 monoclonal antibody has been shown to suppress tumor angiogenesis and progression in a murine xenograft model of ovarian cancer [23, 24]. Thus, targeting VASH2 may also represent a promising therapeutic strategy for ovarian cancer.

Microtubules are a major component of the cytoskeleton, which consists of repeating α‐ and β‐tubulin heterodimers that undergo numerous post‐translational modifications including the tyrosination–detyrosination cycle. Accumulation of detyrosinated tubulin is associated with poorer prognosis in breast cancer and neuroblastoma [25, 26]. Tubulin tyrosination is mediated by tubulin tyrosine ligase, which was first identified and reported in 1980 [27]. However, the specific enzyme responsible for the tubulin detyrosination‐mediated acceleration of tubulin polymerization remains unclear. In 2017, two groups reported that vasohibin family members exhibited TCP activity, catalyzing the C‐terminal tyrosine residue of α‐tubulin [13, 14]. We previously demonstrated that VASH2 knockout cells weakly expressed detyrosinated tubulin [28], whereas the forced expression of VASH1 increased intracellular tubulin detyrosination in this study. These results suggest that both VASH1 and VASH2 exhibit TCP activity and play an important role in microtubule activity in ovarian cancer cells.

Cell division is another important problem area related to microtubules. The cyclin and cyclin‐dependent kinase (CDK) complex has essential functions in the cell cycle. Cyclin B1 is upregulated from the G2 to the early M phase during cell cycle progression. Before forming a complex with CDK1, cyclin B1 induces cell cycle transition from the early M to middle M phase; subsequently, its expression is decreased in the late M phase [29]. In this study, the overexpression of VASH1 increased cyclin B1 expression in ovarian cancer cells. This suggests that VASH1 inhibited the formation of spindle fibers in the late M phase, resulting in the delayed transition of the cell cycle from the middle to late M phase.

PTX stabilizes microtubules by binding β‐tubulin and disrupting the dynamic balance between soluble and polymerized tubulin. PTX impairs the metaphase‐to‐anaphase transition in the M phase, and ultimately induces cell apoptosis [29]. Therefore, PTX shows strong antitumor activity in the middle M phase of the cell cycle. Our results demonstrated that VASH1 overexpression induced strong expression of detyrosinated tubulin and cyclin B1, resulting in significantly augmented PTX chemosensitivity. These findings may be attributed to the increased proportion of cells in the middle M phase. Similar to the present results, cyclin B1 overexpression has been reported to sensitize prostate cancer cells to PTX [30]. In contrast, the overexpression of VASH1 did not significantly affect sensitivity to CDDP, another key chemotherapeutic agent for ovarian cancer, possibly because CDDP targets DNA itself, not microtubules. Similar to VASH1 overexpression, VASH2 knockout induced stronger expression of cyclin B1 and significantly increased PTX sensitivity [28].

Recently, Kobayashi et al. elucidated the relationship between the antiangiogenic effects and TCP activity of VASH1 [31]. VASH1 was found to impair endocytosis and trafficking of VEGF receptor 2 through detyrosination of α‐tubulin in ECs, resulting in decreased VEGF signal transduction and EC migration. VASH1 has multiple functions including increasing chemosensitivity to PTX in tumor cells and abrogating angiogenesis in the tumor microenvironment. Small vasohibin binding protein (SVBP) is essential for efficient secretion of vasohibin family members [32]. VASH1 can be overexpressed using several methods, including recombinant VASH1 protein and administration of VASH1 via an adenovirus vector [18]. However, further studies are necessary to clarify the most efficient method to express VASH1 in the tumor or tumor microenvironment in vivo when considering the application of VASH1 overexpression in clinical settings.

In conclusion, forced expression of VASH1 in ovarian cancer cells was found to enhance TCP activity, increase cyclin B1 expression, and augment PTX chemosensitivity. Ovarian cancer treatment strategies targeting VASH1 can potentiate the effects of conventional chemotherapy by inhibiting angiogenesis and regulating microtubule activity.

## Author Contributions

T.K. and Y.S. performed the experiments and wrote the manuscript. Y.T., K.T., S.T., A.T., and Y.T. provided advice in preparing the manuscript. H.M. and H.F. reviewed and edited the manuscript.

## Ethics Statement

The authors have nothing to report.

## Consent

The authors have nothing to report.

## Conflicts of Interest

The authors declare no conflicts of interest.

## Data Availability

The data that support the findings of this study are available from the corresponding author upon reasonable request.
